# An Intelligent Diagnosis Method for Rotating Machinery Using Least Squares Mapping and a Fuzzy Neural Network

**DOI:** 10.3390/s120505919

**Published:** 2012-05-08

**Authors:** Ke Li, Peng Chen, Shiming Wang

**Affiliations:** 1 Graduate School of Bioresources, Mie University, 1577 Kurimamachiya-cho, Tsu, Mie 514-8507, Japan; 2 College of Engineer Science and Technology, Shanghai Ocean University, No. 999 Hucheng Ring Road, Lingang New City, Shanghai 201306, China; E-Mails: dayanlv@live.cn (K.L.); smwang@shou.edu.cn (S.W.)

**Keywords:** condition diagnosis, least squares mapping, possibility theory, Dempster & Shafer theory, fuzzy neural network

## Abstract

This study proposes a new condition diagnosis method for rotating machinery developed using least squares mapping (LSM) and a fuzzy neural network. The non-dimensional symptom parameters (NSPs) in the time domain are defined to reflect the features of the vibration signals measured in each state. A sensitive evaluation method for selecting good symptom parameters using detection index (DI) is also proposed for detecting and distinguishing faults in rotating machinery. In order to raise the diagnosis sensitivity of the symptom parameters the synthetic symptom parameters (SSPs) are obtained by LSM. Moreover, possibility theory and the Dempster & Shafer theory (DST) are used to process the ambiguous relationship between symptoms and fault types. Finally, a sequential diagnosis method, using sequential inference and a fuzzy neural network realized by the partially-linearized neural network (PLNN), is also proposed, by which the conditions of rotating machinery can be identified sequentially. Practical examples of fault diagnosis for a roller bearing are shown to verify that the method is effective.

## 1. Introduction

In the field of machinery diagnosis, vibration signals are often used for fault detection and state discrimination. Machinery diagnosis depends largely on the feature analysis of vibration signals measured for condition diagnosis, because the signals carry dynamic information about the machine state [1–3]. The vibration signals in different states will show different features, that is to say when plant machinery is in abnormal state, it will output signal sets which correspond to different faults. However, in most cases of condition diagnosis for rotating machinery, the values of symptom parameters calculated from vibration signals for condition monitoring and fault diagnosis are ambiguous. The main reasons for this can be explained as follows: (1) When the rotation speed and load of rotating machinery vary while vibration signals is being measured and a fault is in an early stage, the signal contains strong noise, stronger than the actual failure signal, that may lead to misrecognition of useful diagnostic information; (2) The statistical objectivity of the measured signal cannot always be satisfied because of the measurement techniques and manner of the inspectors [4]. Therefore, it is important to solve the ambiguous problem of fault diagnosis.

Roller bearings are an important part, widely used in rotating machinery. The failure of a rolling bearing may cause the breakdown of a rotating machine, and furthermore, serious consequences may arise due to the failure. Therefore, fault diagnosis of rolling bearings is extremely important for guaranteeing production efficiency and plant safety. Although fault diagnosis of rolling bearings is often artificially carried out using time or frequency analysis of vibration signals, there is a need for a reliable, fast automated diagnosis method thereof. Neural Networks (NN) have potential applications in automated detection and diagnosis of machine failure [5–9]. However, a conventional NN cannot adequately reflect the possibility of ambiguous diagnosis problems, and will never converge, when the symptom parameters, input to the 1st layer of the NN, have the same values in different states [4].

**Figure 1.**
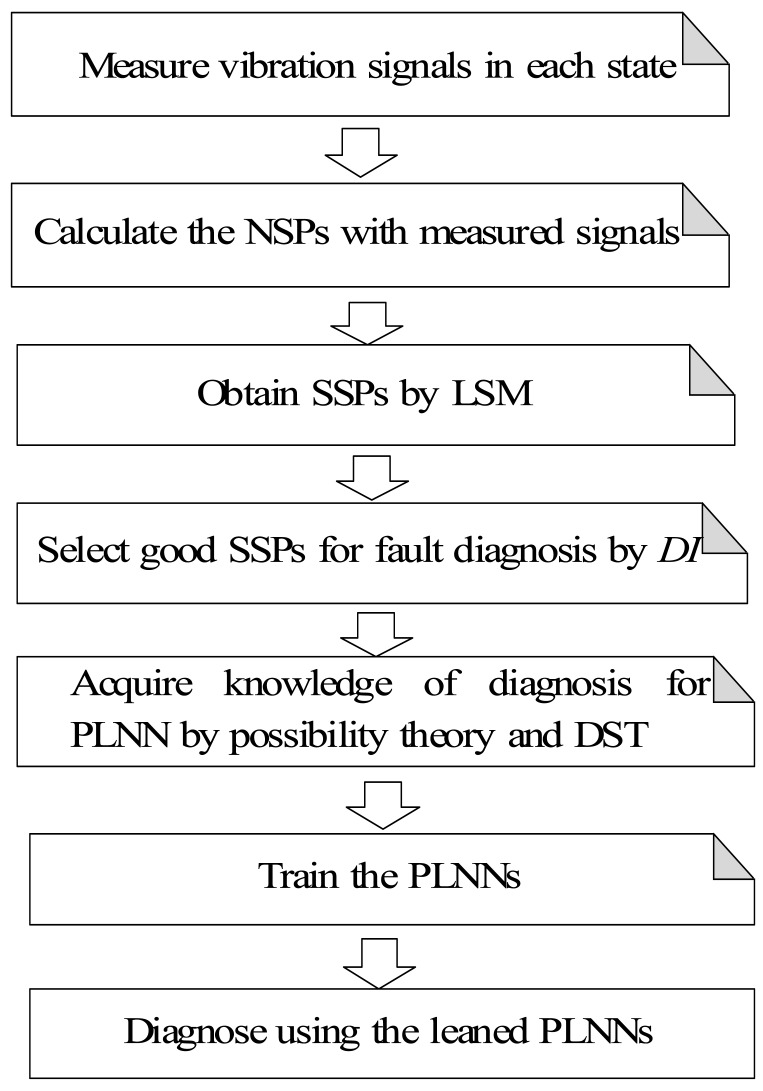
Flowchart of the condition diagnosis.

For the above reasons, this paper proposes a novel condition diagnosis method for rotating machinery developed using LSM and a fuzzy neural network realized by the PLNN. The NSPs in the time domain are defined to reflect the vibration signal features measured in each state. To raise the diagnosis sensitivity of the symptom parameters the SSPs are obtained by LSM. Using statistical theory, a detection index (DI) has also been defined to evaluate the applicability of SSPs. The DI can be used to indicate the fitness of a SSP for the PLNN. A sequential diagnosis approach is also proposed through the PLNN to sequentially identify the types of fault of rotating machinery. Diagnostic knowledge for the PLNN is acquired by possibility theory and the DST for solving the problem of ambiguous fault diagnosis. A practical example of condition diagnosis for a roller bearing verifies that the method is effective. The flowchart of the condition diagnostic procedure proposed in this paper is shown in Figure 1.

## 2. Experimental System for Fault Diagnosis

**Figure 2.**
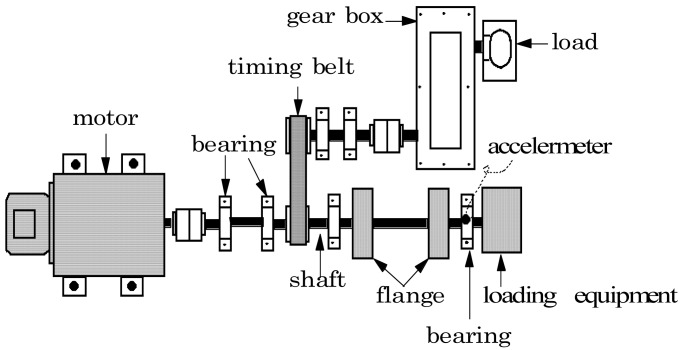
Experimental setup for rolling bearing fault diagnosis.

**Figure 3.**
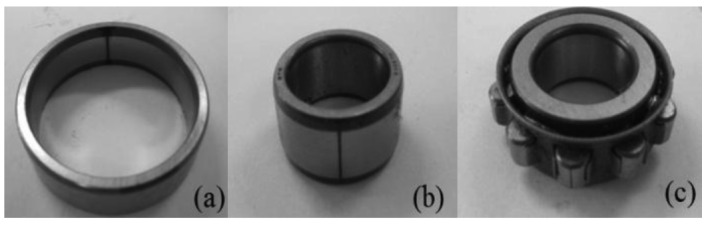
Bearing defects. (**a**) Outer-race defect; (**b**) Inner-race defect; (**c**) Roller defect.

Figure 2 shows the experimental system for the roller bearing fault diagnosis test. The most commonly occurring faults in a roller element bearing are the outer-race defect, the inner-race defect, and the roller element defect. These fault bearings are shown in Figure 3 and were created artificially using a wire-cutting machine. The bearings that were utilized, and specifications of the test bearing, the size of the faults, and other necessary information is listed in Table 1.

**Figure 4.**
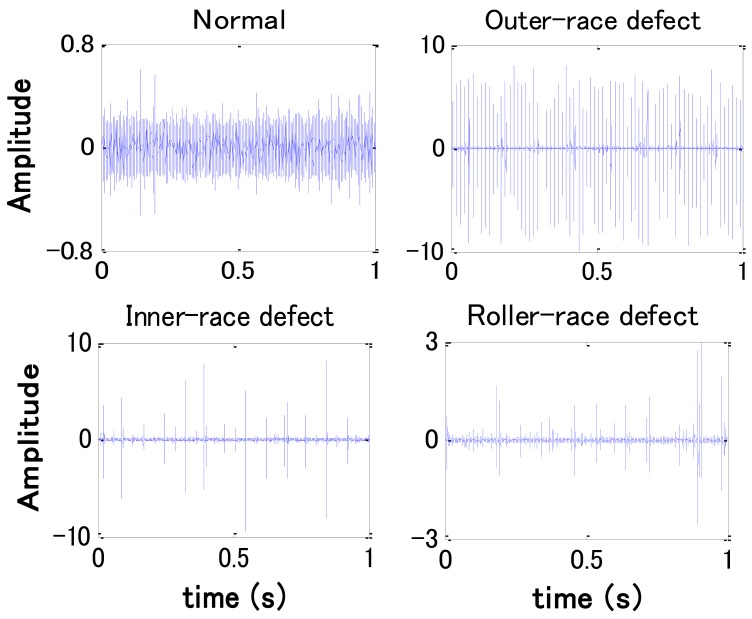
Vibration signals of bearings after filtering.

In this work an accelerometer (PCB MA352A60) with a bandwidth from 5 Hz to 60 kHz and 10 mV/g output was used to measure the vibration signals of the vertical direction in the normal (N), the outer-race defect (O), the inner-race defect (I), and the roller element defect (R) states, respectively. The vibration signals measured by the accelerometer were transformed into a signal recorder (Scope Coder DL750) after being magnified by a sensor signal conditioner (PCB ICP Model 480C02). The original vibration signals in each state are measured at a constant speed (1,500 rpm), and a 150 kg load is also transported on the rotating shaft by the loading equipment (RCS2-RA13R) while the vibration signals are being measured. A high-pass filter with a 5 kHz cut-off frequency was used to cancel noise in the vibration signals for fault diagnosis. Examples of vibration signals measured in each state after filtering are shown in Figure 4. The sampling frequency of the signal measurement is 50 kHz, and the sampling time is 20 s.

## 3. Non-Dimensional Symptom Parameters and Sensitivity Evaluation

### 3.1. Non-Dimensional Symptom Parameters for Fault Diagnosis

When a computer is used for condition diagnosis of plant machinery, symptom parameters (SPs) are required to express the information indicated by a signal measured for diagnosing machinery faults. A good symptom parameter can correctly reflect states and the condition trends of plant machinery [10–12]. Many symptom parameters have been defined in the pattern recognition field [13]. Here, eight NSPs in the time domain, commonly used for the fault diagnosis of plant machinery, are considered:

(1) $$P_{1}=\frac{\sigma }{\bar{x}}$$

(2) $$P_{2}=\frac{\sum_{i=1}^{N}x_{i}^{2}}{N\sigma^{2}}$$

(3) $$P_{3}=\frac{\left|\sum_{i=1}^{N}{\left(x_{i}-\bar{x}\right)}^{3}\right|}{N\sigma^{3}}$$

(4) $$P_{4}=\frac{\sum_{i=1}^{N}{\left(x_{i}-\bar{x}\right)}^{4}}{N\sigma^{4}}$$

where *x_i_* is digital data of vibration signal. *x̄* is the mean value of *x_i_*, $\bar{x}=\frac{\sum_{i=1}^{N}x_{i}}{N}$ *σ* is the standard deviation of x_i_, $\sigma =\sqrt{\frac{\sum_{i=1}^{N}{\left(x_{i}-\bar{x}\right)}^{2}}{N-1}}$. *N* is the number of *x_i_*:

(5) $$P_{5}=\frac{\left|\sum_{i=1}^{N_{p}}{\left(x_{pi}-\bar{x_{p}}\right)}^{3}\right|}{N_{p}{\sigma_{p}}^{3}}$$

(6) $$P_{6}=\frac{\left|\sum_{i=1}^{N_{p}}{\left(x_{pi}-\bar{x_{p}}\right)}^{4}\right|}{N_{p}{\sigma_{p}}^{4}}$$

where *x_pi_* is the peak value of *x_i_, x̄_p_* and *σ_p_* are the mean value and standard deviation of *x_pi_*, respectively. *N_p_* is the number of *x_pi_*:

(7) $$P_{7}=\frac{\left|\sum_{i=1}^{N_{v}}{\left(x_{vi}-\bar{x_{v}}\right)}^{3}\right|}{N_{v}{\sigma_{v}}^{3}}$$

(8) $$P_{8}=\frac{\left|\sum_{i=1}^{N_{v}}{\left(x_{vi}-\bar{x_{v}}\right)}^{4}\right|}{N_{v}{\sigma_{v}}^{4}}$$

where *x_vi_* is the valley value of *x_i_*. *x̄_v_* and *σ_v_* are the mean value and standard deviation of *x_vi_*, respectively. *N_v_* is the number of *x_vi_*.

### 3.2. Detection Index

Supposing that *x*_1_ and *x*_2_ are values of a symptom parameter (SP) calculated from the signals measured in state 1 and state 2, respectively, and conforming respectively to the normal distributions N(*μ*_1_,*σ*_1_) and N(*μ*_2_,*σ*_2_). Here, μ and σ are the average and the standard deviation of the SP. The larger the value of |x_2_−x_1_| is, the higher the sensitivity of distinguishing the two states by the SP. Because *z* = *x*_2_ − *x*_1_ also conforms to the normal distribution N(*μ*_2_ − *μ*_1_,*σ*_1_ + *σ*_2_), there is the following density function about *z*:

(9) $$f(z)=\frac{1}{\sqrt{2\pi (\sigma_{1}^{2}+\sigma_{2}^{2})}}\exp\{\frac{{\left\{z-(\mu_{2}-\mu_{1})\right\}}^{2}}{2(\sigma_{1}^{2}+\sigma_{2}^{2})}\}$$

where, *μ*_2_ ≥ *μ*_1_ (the same conclusion can be drawn when *μ*_1_ ≥ *μ*_2_). The probability can be calculated with the following formula:

(10) $$P_{0}=\int_{-\infty }^{0}f(z)d_{z}$$

where, 1-*P*_0_ is called the “Discrimination Rate (DR)”. With the substitution:

(11) $$\mu =\frac{z-(\mu_{2}-\mu_{1})}{\sqrt{\sigma_{1}^{2}+\sigma_{2}^{2}}}$$

into Equations (9) and (10), the *P*_0_ can be obtained by:

(12) $$P_{0}=\frac{1}{\sqrt{2\pi }}\int_{-\infty }^{-DI}\exp(-\frac{\mu^{2}}{2})d_{\mu }$$

where, the DI (Detection Index) is calculated by:

(13) $$DI=\frac{\mu_{2}-\mu_{1}}{\sqrt{\sigma_{1}^{2}+\sigma_{2}^{2}}}\;\text{or}\;DI=\frac{\bar{x_{2}}-\bar{x_{1}}}{\sqrt{\sigma_{1}^{2}+\sigma_{2}^{2}}}$$

It is obvious that the larger the value of the DI, the larger the value of the “Discrimination Rate (DR = 1 − *P*_0_)” will be, and therefore, the better the SP will be. Thus, the DI can be used as the index of the quality to evaluate the distinguishing sensitivity of the SP. The number of symptom parameters used for the diagnosis and fault types are *M* and *N*, respectively, and the synthetic detection index (SDI) is defined as follows:

(14) $$\text{SDI}=\sum_{i=1}^{N-1}\sum_{j=i+1}^{N}\sum_{k=1}^{M}\frac{\left|\mu_{ik}-\mu_{jk}\right|}{\sqrt{\sigma_{ik}^{2}+\sigma_{jk}^{2}}}$$

Table 2 lists the diagnosis sensitivity standard for condition diagnosis.

## 4. Synthesizing Symptom Parameter by Least Squares Mapping

In order to raise the diagnosis sensitivity of the symptom parameter, a method for obtaining the new synthetic symptom parameter is proposed as follows. The least squares mapping (LSM) technique aims to increase class separability and consists of the transformation of pattern vectors around arbitrary pre-selected points in the **R**^**C**^ space (where *C* is the number of states), called the decision space, in such a way that the least squares transformation error is minimized [14,15]. In this section, we propose a method used to raise the diagnosis sensitivity by projecting the SPs into discrimination space using least squares mapping. The type number of SPs (**Y_k_**) is *K*, and the category number of states is *M*. In the coordinate space of *K* dimension, the endpoint of the vector **Y_ij_** expresses state*i*. **Y_ij_** is shown as follows:

(15) $${\text{Y}}_{\text{ij}}={\left\{y_{ij1},y_{ij2},\cdots ,y_{ijK}|i=1\sim M,j=1\sim N\right\}}^{T}$$

where, *N* is the number of SPs, and the number of SPs in each state is same.

**Y_ij_** can be projected into a new space **L**, and the new vector **L_ij_** in the space **L** can be calculated as follows:

(16) $${\text{L}}_{\text{ij}}=\text{A}{\text{Y}}_{\text{ij}}$$

where:

(17) $${\text{L}}_{\text{ij}}={\left\{l_{ij1},l_{ij2},\cdots ,l_{ijK}|i=1\sim M,j=1\sim N\right\}}^{T}$$

The transformation matrix **A** is defined by means of minimizing the least squares error (***ε***) between vectors **L_ij_** and **V_i_** for all states, where **V_i_** is an arbitrary selected vector point in the **L** space. The selection of vector **V_i_** is critical to enhance sensitiveness of the synthetic symptom parameter. In the present work, **V_i_** is determined as a unit orthogonal vector by experience.

**Figure 5.**
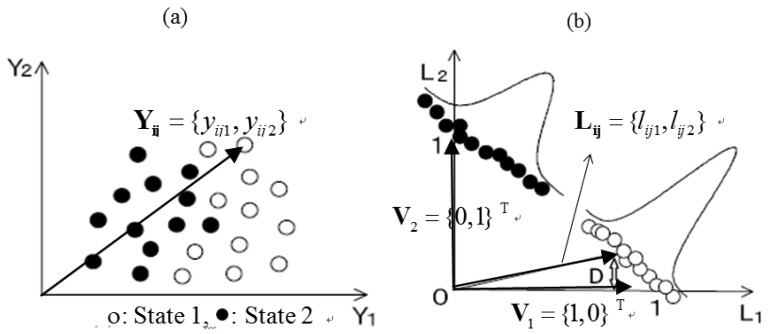
Projected example by the LSM (**a**) before projection; (**b**) after projection.

Figure 5 shows an illustration of the projection by the LSM, where *K* = 2 and *M* = 2. Namely, the two states (state 1 and state 2) should be classified using two SP series.

The error vector is:

(18) $$ɛ=\frac{1}{N}\sum_{j=1}^{N}{\left\|{\text{L}}_{\text{ij}}-{\text{V}}_{\text{i}}\right\|}^{2}$$

***ε*** minimization is performed by solving the following equation over **A**:

(19) $$\nabla_{A}ɛ=0$$

which, in conjunction with (18), leads to:

(20) $$\text{A}=\left[\sum_{j=1}^{N}\left\{{\text{V}}_{\text{i}}{\text{Y}}_{\text{ij}}^{\prime }\right\}\right]\;{\left[\sum_{j=1}^{N}\left\{{\text{Y}}_{\text{ij}}{\text{Y}}_{\text{ij}}^{\prime }\right\}\right]}^{-1}$$

When *M* ≥ 2, A is decided as follows:

(21) $$\text{A}=\left[\sum_{i=1}^{M}\sum_{j=1}^{N}\left\{{\text{V}}_{\text{i}}{\text{Y}}_{\text{ij}}^{\prime }\right\}\right]\;{\left[\sum_{i=1}^{M}\sum_{j=1}^{N}\left\{{\text{Y}}_{\text{ij}}{\text{Y}}_{\text{ij}}^{\prime }\right\}\right]}^{-1}$$

For diagnosis, the new synthetic symptom parameter can be obtained as follows:

(22) SSP=A⋅SP

where SP indicates symptom parameter (here P_1_∼P_8_).

According to the projected results shown in Figure 5(b), the points in state 1 and state 2 are congregated to vector **V_1_** and **V_2_**, respectively. The two states in the space **L** can be distinguished more easily than in the space **Y**.

To explain the efficiency of the LSM method, some examples are given. In the present example, we used two symptom parameters (P_1_ and P_2_) to distinguish the inner race defect (I) and roller element defect (R) states of the bearing. SSP_1_ and SSP_2_ are the new synthetic parameter obtained by the LSM. Tables 3 and 4 show the parameters and the values of the DI and the DR before projection and after projection by the LSM, respectively. According to those examples, the states can be clearly distinguished by the SSPs. It is obvious that the sensitivity of the SSPs obtained by the LSM is higher than the original SPs. In Tables 3 and 4, μ_p1_, μ_p2_, μ_ssp1_ and μ_ssp2_ are the mean values of P_1_, P_2_, SSP_1_ and SSP_2_, respectively. σ_p1_, σ_p2_, σ_ssp1_ andσ_ssp2_ are the standard deviations of P_1_, P_2_, SSP_1_ and SSP_2_, respectively.

## 5. Sequential Diagnosis Method Based on Fuzzy Inference and Dempster & Shafer Theory

### 5.1. Sequential Condition Diagnosis Approach

**Figure 6.**
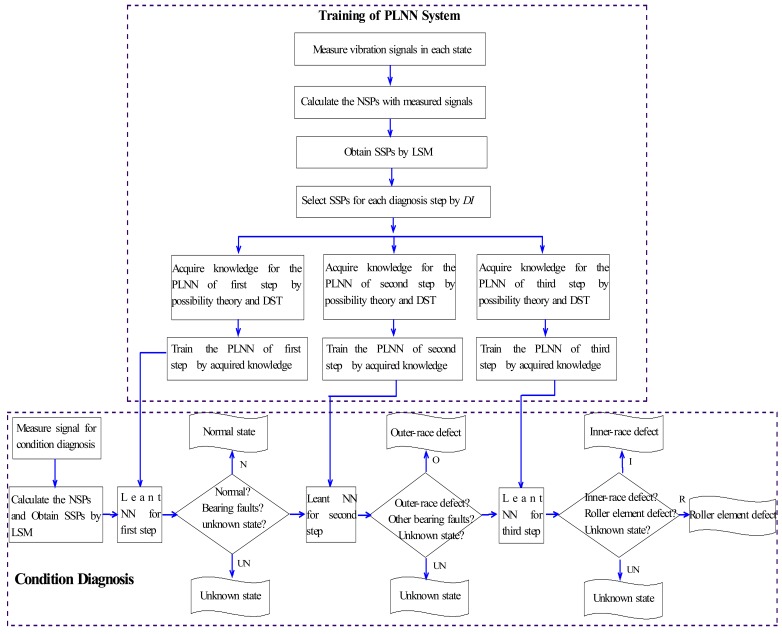
Flowchart of sequential condition diagnosis.

In many cases of condition diagnosis, symptom parameters are defined to reflect the features of vibration signals measured in each state in order to diagnose faults. However, it is difficult to find one symptom parameter or a few symptom parameters that can identify all of the faults simultaneously. However, the symptom parameters for identification of two states are easy to identify [16]. In order to solve these problems, a sequential diagnosis method is proposed. In the first step, the normal state (N) can be distinguished from abnormal states using the corresponding possibility of the symptom parameter. In the second step, the outer-race defect (O) can be distinguished from the other abnormal states using the corresponding possibility of the symptom parameter. In the last step, the inner-race defect (I) and the roller element defect (R) states can be distinguished using the corresponding possibility of the symptom parameter. Figure 6 shows the flowchart of sequential condition diagnosis proposed in this study.

As mentioned in the Section 3.2, the larger the value of the DI, the better the SP will be. Therefore, the two best SSPs that have the high sensitivity at each diagnostic step are selected by the DI. As an example, parts of the DI values of each SSP and the selection results are shown in Table 5. In the first step, SSP_1_ and SSP_5_ can distinguish the normal (N) and the abnormal states (O, I and R) more easily than the other SSPs. Because all of DI values of SSP_1_ and SSP_5_ for distinguishing these states are larger than those of the other SSPs. Similarly, the SSPs for other diagnostic steps can also be selected. The other selected results of the SSPs are, SSP_1_ and SSP_5_ for the second step, and SSP_1_ and SSP_2_ for the last step, respectively. All of those DIs are larger than 2.12, and therefore all of the distinction rates approach 98.5%.

### 5.2. Fuzzy Inference by Possibility Theory

In most cases of condition diagnosis for rotating machinery, knowledge of distinguishing faults is ambiguous, because the definite relationships between symptom parameters and fault types, even for a single fault, cannot be easily identified. The values of symptom parameters calculated from vibration signals for fault diagnosis are also ambiguous because of the dispersion in the same state. Therefore, it is necessary to solve the ambiguous problem of fault diagnosis and to express uncertainty about the interpretation of the observable.

Possibility theory is a mathematical theory for dealing with certain types of uncertainty and is an alternative to probability theory. Zadeh first introduced possibility theory in 1978 as an extension of his theory of fuzzy sets and fuzzy logic [17]. Dubois and Prade further contributed to its development [18,19]. Recently, possibility theory has been used for fault diagnosis [16,20]. More details about possibility theory were introduced in references [21–23]. In the present work, possibility theory is applied to solving the ambiguous relationship between the symptom parameters and fault types.

For fuzzy inference, membership functions of SP are necessary. These can be obtained from probability density functions of the symptom parameters using possibility theory. When the probability density function of symptom parameters conforms to the normal distribution, it can be changed to a possibility function *P(x_i_)* using the following formula:

(23) $$P(x_{i})=\sum_{k=1}^{N}\min\left\{\lambda_{i},\lambda_{k}\right\}$$

where *λ*_i_ and *λ*_k_ can be calculated as follows:

(24) $$\lambda_{i}=\int_{x_{i}}^{x_{i}}\frac{1}{\sigma \sqrt{2\pi }}\exp\left\{-\frac{{\left(x-\bar{x}\right)}^{2}}{2\sigma^{2}}\right\}dx$$

(25) $$\lambda_{k}=\int_{k_{i-1}}^{k_{i}}\frac{1}{\sigma \sqrt{2\pi }}\exp\left\{-\frac{{\left(x-\bar{x}\right)}^{2}}{2\sigma^{2}}\right\}dx$$

where σ and x̄ are the standard deviation and the mean value of the SP, respectively, and *x* = *x̄* − 3*σ* ∼ *x̄* + 3*σ*.

**Figure 7.**
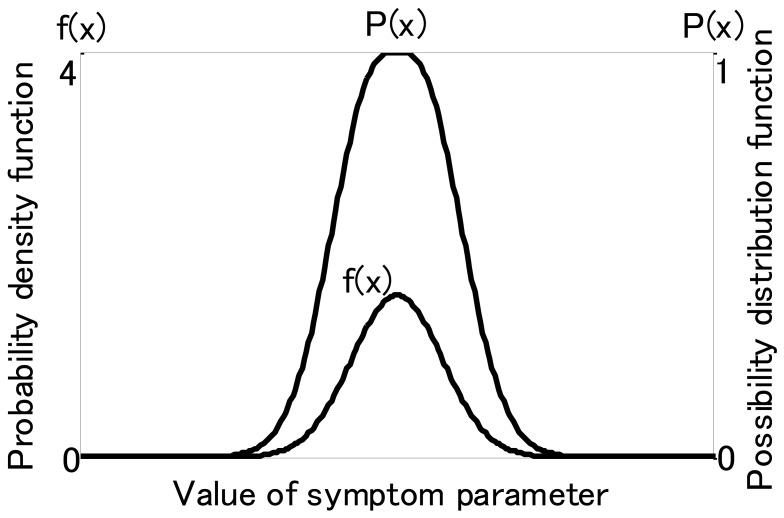
Possibility function and the probability density function.

**Figure 8.**
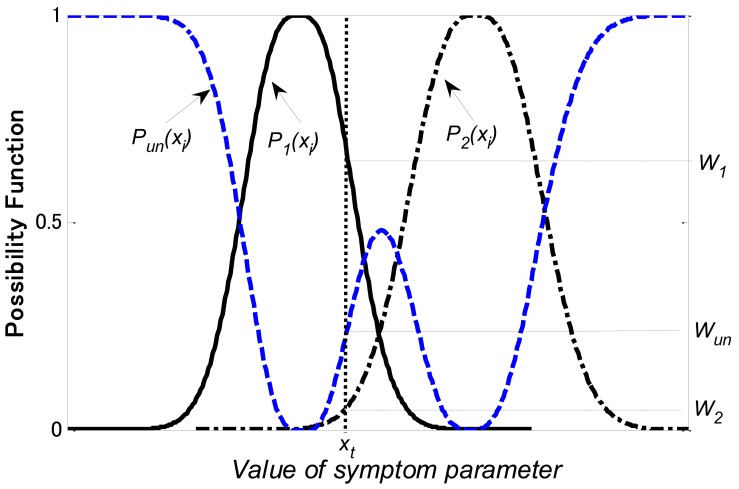
Matching examples of possibility function.

Figure 7 shows an illustration of the possibility function and the probability density function. Figure 8 shows the matching examples of possibility function. In the present example, we used the symptom parameter (*x_i_*) to distinguish state1, state 2 and unknown state. *P_1_*(*x_i_*) and *P_2_*(*x_i_*) are possibility functions for state 1 and state 2, respectively. The possibility function of unknown state can be calculated as follows,

(26) $$P_{\text{un}}(x_{i})=\max\left\{0,1-\left[P_{1}(x_{i})+P_{2}(x_{i})\right]\right\}$$

If *x_t_* is the symptom parameter calculated from the data in the state to be diagnosed, the matching degrees with a relevant level are calculated as follows:

(27) $$\text{State}\;1\;\text{level}:W_{1}=P_{1}(x_{i})\cap x_{t}$$

(28) $$\text{State}\;2\;\text{level}:W_{2}=P_{2}(x_{i})\cap x_{t}$$

(29) $$\text{Unknown state level}:W_{un}=P_{un}(x_{i})\cap x_{t}$$

Where *W_1_, W_2_* and *W_un_* express the possibilities of state 1, state 2 and unknown state, respectively. These degrees are normalized by

(30) $$W_{1}+W_{2}+W_{\text{un}}=1$$

Fuzzy systems rely on a set of rules. In this study, to correctly and effectively identify the condition and the fault type of rotating machinery, we have obtained the following “if-then” rules for condition diagnosis.

Rule 1: *if x_i_* < *x̄*_1*i*_ − 3*σ*_1_ *and x_i_* < *x̄*_2*i*_ − 3*σ*_2_ *then W*_1_ = 0, *W*_2_ = 0, *W_un_* = 1;
Rule 2: *if x_i_* > *x̄*_1*i*_ + 3*σ*_1_ *and x_i_* > *x̄*_2*i*_ + 3*σ*_2_ *then W*_1_ = 0, *W*_2_ = 0, *W_un_* =1;
Rule 3: *if x̄*_1*i*_ − 3*σ*_1_ ≤ *x_i_* ≤ *x̄*_1*i*_ + 3*σ*_1_*then* 0 ≤ *W*_1_ ≤ 1, 0 ≤ *W*_2_ ≤ 1, 0 ≤ *W_un_* < 1;
Rule 4: *if x̄*_2*i*_ − 3*σ*_2_ ≤ *x_i_* ≤ *x̄*_2*i*_ + 3*σ*_2_ *then* 0 ≤ *W*_1_ ≤ 1, 0 ≤ *W*_2_ ≤ 1, 0 ≤ *W_un_* < 1;

where *x̄*_1*i*_ and *x̄*_2*i*_ are mean values of symptom parameter *x_i_* in states 1 and 2, respectively; *σ*_1_ and *σ*_2_ are standard deviations of symptom parameter *x_i_* in states 1 and 2, respectively. In the rules 3 and 4, the possibilities *W*_1_, *W*_2_ and *W_un_* can be obtained by Equations (27–29), respectively.

### 5.3. Dempster & Shafer Theory

Dempster & Shafer theory (DST) provides a rational inference mechanism for the combination relation in the diagnosis problems with uncertainty [24–28]. To obtain the results of the condition diagnosis by fuzzy inference, the combination functions of the symptom parameters are necessary. In the present work, the combining possibility function of the symptom parameters (SP*_i_* and SP*_j_*) can be obtained by the Dempster & Shafer theory (DST).

Supposing *W_i_*(*A_m_*) is possibility of SP*_i_* in state *A_m_; W_j_*(*A_k_*) is possibility of SP*_j_* in state *A_k_*, here, *A_m_* and *A_k_* are state sets, and *m* = *k* = {1,2,…n}. *W(S)*^′^ is the combination possibility function of SP*_i_* and SP*_j_*, and *S* ∈ *A_m_* and *A_k_*. Thus, *W(S)*^′^can be obtained by:

(31) $$W{\left(S\right)}^{\prime }=\frac{\sum_{A_{m}\cap A_{k}=S}W_{i}(A_{m})\cdot W_{j}(A_{k})}{1-\sum_{A_{m}\cap A_{k}=\Phi }W_{i}(A_{m})\cdot W_{j}(A_{k})}$$

where Φ expresses an empty set.

As mentioned above, the combination possibility functions of SSPs in each sequential diagnosis step are obtained as follows. In the first step of the sequential diagnosis, the normalized combination possibility functions of the normal state possibility *W(N)*′, bearing fault state possibility *W(B)*′ and unknown state possibility *W(U)*′ can be obtained through the possibilities *W_i_*(…) and *W_j_*(…) of *SSP_i_* and *SSP_j_* (here *i* = 1, and *j* = 5), respectively, as follows:

(32) $$W{\left(N\right)}^{\prime }=\frac{W_{i}(N)\cdot W_{j}(N)+W_{i}(N)\cdot W_{j}(U)+W_{i}(U)\cdot W_{j}(N)}{1-W_{i}(N)\cdot W_{j}(B)-W_{i}(B)\cdot W_{j}(N)}$$

(33) $$W{\left(B\right)}^{\prime }=\frac{W_{i}(B)\cdot W_{j}(B)+W_{i}(B)\cdot W_{j}(U)+W_{i}(U)\cdot W_{j}(B)}{1-W_{i}(N)\cdot W_{j}(B)-W_{i}(B)\cdot W_{j}(N)}$$

(34) $$W{\left(U\right)}^{\prime }=\frac{W_{i}(U)\cdot W_{j}(U)}{1-W_{i}(N)\cdot W_{j}(B)-W_{i}(B)\cdot W_{j}(N)}$$

where *W_i_*(*N*), *W_i_*(*B*) and *W_i_*(*U*) are possibilities of normal state (N), bearing fault state (B) and unknown state (U) obtained by *SSP_i_*, respectively. *W_j_*(*N*), *W_j_*(*B*) and *W_j_*(*U*) are possibilities of normal state (N), bearing fault state (B) and unknown state (U) obtained by *SSP_j_*, respectively.

In the second step of the sequential diagnosis, the normalized combination possibility functions of the outer-race defect possibility *W(O)*′, other bearing defects possibility *W(IR)*′, and the unknown state possibility *W(U)*′can be obtained through the possibilities *W_i_*(…) and *W_j_*(…)of *SSP_i_* and *SSP_j_* (here, *i* = 1, *j* = 5), respectively, as follows:

(35) $$W{\left(O\right)}^{\prime }=\frac{W_{i}(O)\cdot W_{j}(O)+W_{i}(O)\cdot W_{j}(U)+W_{i}(U)\cdot W_{j}(O)}{1-W_{i}(O)\cdot W_{j}(IR)-W_{i}(IR)\cdot W_{j}(N)}$$

(36) $$W{\left(IR\right)}^{\prime }=\frac{W_{i}(IR)\cdot W_{j}(IR)+W_{i}(IR)\cdot W_{j}(U)+W_{i}(U)\cdot W_{j}(IR)}{1-W_{i}(O)\cdot W_{j}(IR)-W_{i}(IR)\cdot W_{j}(N)}$$

(37) $$W{\left(U\right)}^{\prime }=\frac{W_{i}(U)\cdot W_{j}(U)}{1-W_{i}(O)\cdot W_{j}(IR)-W_{i}(IR)\cdot W_{j}(O)}$$

where *W_i_(O), W_i_(IR)* and *W_i_(U)* are possibilities of outer-race defect (O), other bearing defects (IR) and unknown state (U) obtained by SSP_i_, respectively. *W_j_(O), W_j_(IR)* and *W_j_(U)* are possibilities of outer-race defect (O), other bearing defects (IR) and unknown state (U) obtained by *SSP_j_*, respectively.

The last step of the sequential diagnosis, the normalized combination possibility function of the inner race defect possibility *W(I)*′, rolling element defect possibility *W(R)*′, and unknown state possibility *W(U)*′ can be obtained through the possibilities *W_i_*(…) and *W_j_*(…) of *SSP_i_* and *SSP_j_* (here, *i* = 1, *j* = 2), respectively, as follows:

(38) $$W{\left(I\right)}^{\prime }=\frac{W_{i}(I)\cdot W_{j}(I)+W_{i}(I)\cdot W_{j}(U)+W_{i}(U)\cdot W_{j}(I)}{1-W_{i}(I)\cdot W_{j}(R)-W_{i}(R)\cdot W_{j}(I)}$$

(39) $$W{\left(R\right)}^{\prime }=\frac{W_{i}(R)\cdot W_{j}(R)+W_{i}(R)\cdot W_{j}(U)+W_{i}(U)\cdot W_{j}(R)}{1-W_{i}(I)\cdot W_{j}(R)-W_{i}(R)\cdot W_{j}(I)}$$

(40) $$W{\left(U\right)}^{\prime }=\frac{W_{i}(U)\cdot W_{j}(U)}{1-W_{i}(I)\cdot W_{j}(R)-W_{i}(R)\cdot W_{j}(I)}$$

where *W_i_(I), W_i_(R)* and *W_i_(U)* are possibilities of inner race defect (I), rolling element defect (R) and unknown state (U) obtained by SSP_i_, respectively. W_j_(I), W_j_(R) and W_j_(U) are possibilities of inner race defect (I), rolling element defect (R) and unknown state (U) obtained by *SSP_j_*, respectively.

## 6. Fuzzy Neural Network for Fault Diagnosis

The main mathematic symbols used in Section 6 are:

*N_m_: the neuron number of the m-th layer of an NN, m* = 1 *to M*.

$X^{\left(1\right)}=\left\{X_{i}^{\left(i,j\right)}\right\}$: *the pattern input to the 1st layer. Here*, $X_{i}^{\left(1,j\right)}$ *is the value input to the j-th neuron in the input (1st) layer, i* = 1 *to P, j* =1 *to N*_1_.

$X^{\left(M\right)}=\{{X_{i}}^{\left(M,k\right)}\}$: *the training (teaching) data for the last layer (M-th layer). Here*, $X_{i}^{\left(M,k\right)}$ *is the output value of the k-th neuron in the output (M-th) layer; k* = 1 *to N*_M_.

$X^{(1)*}=\{X_{i}^{(1,j)*}\}$ *and* $X^{(M)*}=\left\{X_{(M,k)*}^{1}\right\}$: *new data that has not yet been learnt by the NN*.

$X_{i}^{\left(m,t\right)}$: *the value of the t-th neuron in the hidden (m-th) layer; t* =1 *to N_M_*.

$W_{uv}^{\left(m\right)}$: *the weight between the u-th neuron in the m-th layer and the v-th neuron in the* (*m*+*1*)-*th layer, m* =1 *to M* − 1;*u* = 1 *to N_m_; v* =1 *to N*_*m*+1_.

The fuzzy neural network is applied to diagnose the fault types of a rolling bearing by the sequential diagnosis algorithm, and realized with a developed back propagation neural network called as “the partially-linearized neural network” (PLNN). A back propagation neural network is only used for training the data, and the PLNN is used for testing the learned NN. Here, the basic principle of the PLNN for the fault diagnosis is described as follows.

The neuron number of the *m*-th layer of an NN is *N_m_*. The set $X^{\left(1\right)}=\{{X_{i}}^{\left(1,j\right)}\}$ represents the pattern input to the 1st layer and the set $X^{\left(M\right)}=\{{X_{i}}^{\left(M,k\right)}\}$ is the training data for the last layer (*M*-th layer). Here, *i* = 1 *to P, j* = 1 *to N*_1_, *k* = 1 *to N*_M_ and, $X_{i}^{\left(1,j\right)}$: the value input to the *j*-th neuron in the input (1st) layer; $X_{i}^{\left(M,k\right)}$ the output value of the *k*-th neuron in the output (*M*-th) layer, *k* =1 *to N_M_*.

**Figure 9.**
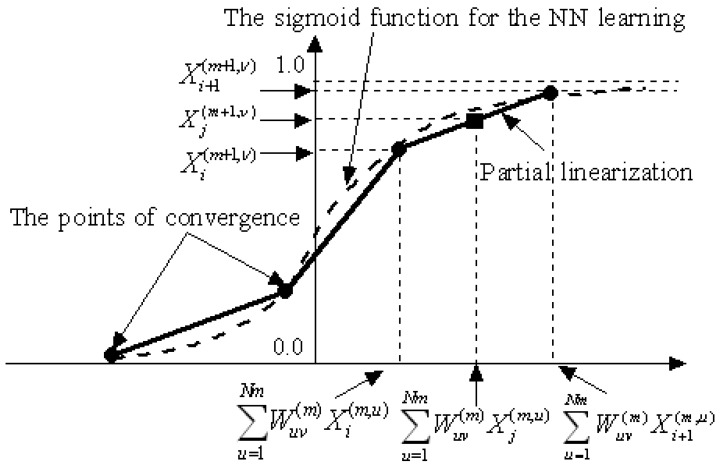
The partial linearization of the sigmoid function.

Even if the NN converges by learning *X*^(1)^ and *X^(M)^*, it cannot adequately deal with the ambiguous relationship between the new *X*^(1)^* and X^(M)^*, which has not been learnt. In order to predict X^(M)^* according to the probability distribution of *X*^(1)^*, partial linear interpolation of the NN is introduced as shown in Figure 9.

In the NN that has converged with the data *X*^(1)^ and *X^(M)^*, the following symbols are used:

$X_{i}^{\left(m,t\right)}$ the value of the *t*-th neuron in the hidden (*m*-th) layer; *t* =1 *to N_m_*.

$W_{uv}^{\left(m\right)}$: the weight between the *u*-th neuron in the *m*-th layer and the *v*-th neuron in the (*m*+*1*)-th layer, *m* =1 *to M;u* = 1 *to N_m_*: *v* = 1 *to N*_*m*+1_.

If all these values are memorized by the computer, when new values $X_{j}^{(1,u)*}$ ( ${X_{j}}^{\left(1,u\right)}<X_{j}^{(1,u)*}<{X_{j+1}}^{\left(1,u\right)}$) are input into the first layer, the predicted value of the *v*-th neuron (*v*=*1 to N_m_*) in the (*m*+*1*)-th layer (*m* = 1 *to M - 1*) can be estimated by:

(41) $$X_{j}^{\left(m+1,\nu \right)}=X_{i+1}^{\left(m+1,\nu \right)}-\frac{\{\sum_{u=1}^{Nm}W_{uv}^{\left(m\right)}(X_{i+1}^{\left(m,u\right)}-X_{j}^{\left(m,u\right)})\}(X_{i+1}^{\left(m+1,v\right)}-X_{i}^{\left(m+1,v\right)})}{\sum_{u=1}^{Nm}W_{uv}^{\left(m\right)}(X_{i+1}^{\left(m,u\right)}-X_{i}^{\left(m,u\right)})}$$

Using the operation above, the sigmoid function is partially linearized, as shown in Figure 9. If a function must be learned, the PLNN will learn the points indicated by the ● symbols shown in Figure 8. When new data (*s_1_*′, *s_2_*′) are input into the converged PLNN, the values depicted by the ■ symbols corresponding to the data (*s_1_*′, *s_2_*′) will quickly be identified as *P_e_*. Thus, the PLNN can be used to deal with ambiguous diagnosis problems.

**Figure 10.**
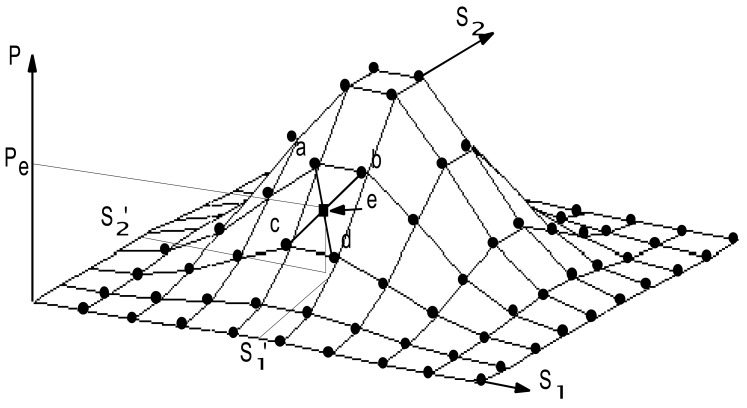
Interpolation by the PLNN.

As shown in Figure 10, the new data (*s_1_*′, *s_2_*′) input into the converged PLNN, and which are not learnt by the PLNN for recognizing, must satisfy the following condition:

(42) $$S_{1(\min)}<S_{1}\prime <S_{1(\max)}\;\text{and}\;S_{2(\min)}<S_{2}\prime <S_{2(\max)}$$

where *S*_1(min)_, *S*_2(min)_ and *S*_1(max)_, *S*_2(max)_ are the minimum values and the maximum values of *S*_1_ and *S*_2_, respectively, which have been learned by the PLNN. Therefore, in this work, the values (P_i_* and P_j_*) of symptom parameters input to the PLNN for fault diagnosis must satisfy the following condition:

(43) $$P_{i(\min)}<{P_{1}}^{*}<P_{i(\max)}\;\text{and}\;P_{j(\min)}<{P_{1}}^{*}<P_{j(\max)}$$

where *P*_*i*(min)_, *P*_*j*(min)_ and *P*_*i*(max)_, *P*_*j*(max)_ are the minimum values and the maximum values of *P_i_* and *P_j_*, respectively.

## 7. Diagnosis and Verification

**Figure 11.**
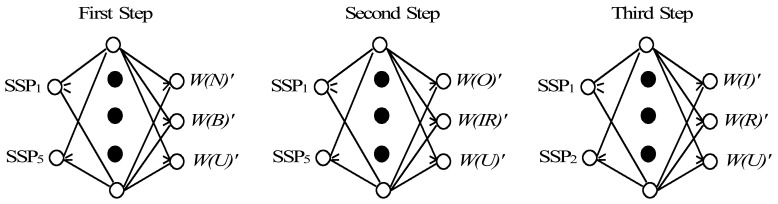
Partially-linearized neural network for condition diagnosis.

Figure 11 shows the PLNNs constructed for the condition diagnosis, which consists of the first layer, the hidden layer and the last layer. The SSPs selected by DI are input into the neurons in the first layer. The number of neurons in hidden layer is eighty. The outputs in the last layer are *W(N)*′, *W(B)*′, *W(O)*′, *W(IR)*′, *W(I)*′, *W(R)*′and *W(U)*′, which mean the possibility grades of normal state, bearing fault state, outer race defect state, other bearing defect, inner race defect, rolling element defect and unknown states, respectively.

**Figure 12.**
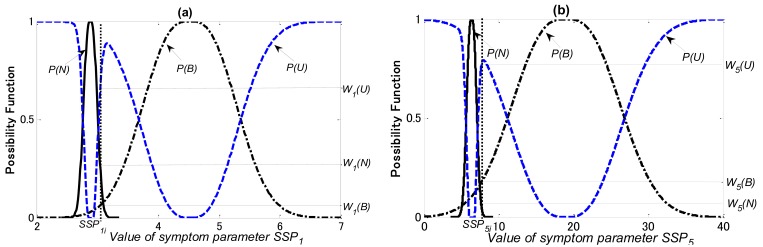
Possibility functions of (**a**) SSP_1_ and (**b**) SSP_5_for first diagnostic step.

**Figure 13.**
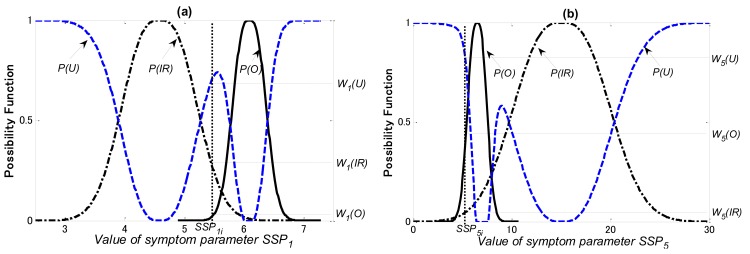
Possibility functions of (**a**) SSP_1_ and (**b**) SSP_5_for second diagnostic step.

**Figure 14.**
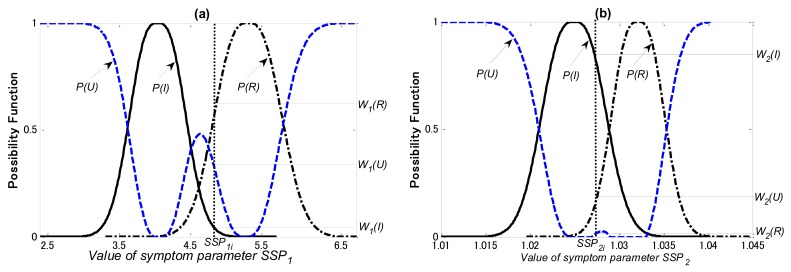
Possibility functions of (**a**) SSP_1_ and (**b**) SSP_2_ for third diagnostic step.

In this study, the diagnosis knowledge for training of the PLNN is acquired by the possibility theory and the Dempster & Shafer theory (DST). The possibility functions of the SSPs used for each diagnostic step, as examples, are shown in Figures 12–14, respectively.

In Figure 12 *P(N), P(B)* and P*(U)* are the possibility functions of the normal, bearing defect and the unknown states, respectively. Using the matching method explained in Section 5.2, *W_1_(N), W_1_(B)* and *W_1_(U)* that the possibilities of *SSP_1_* in the normal, the bearing defect and the unknown states can be obtained, respectively; *W_5_(N), W_5_(B)* and *W_5_(U)* that the possibilities of *SSP_5_* in the normal, the bearing defect and the unknown states can also be obtained, respectively.

In Figure 13 *P(O), P(IR)* and P*(U)* are the possibility functions of the outer-race defect, other bearing faults (the rolling element defect and the inner-race defect), and the unknown states, respectively. Using the matching method explained in Section 5.2, *W_1_(O), W_1_(IR)* and *W_1_(U)* that the possibilities of *SSP_1_* in the outer-race defect, other bearing faults and the unknown states can be obtained, respectively; *W_5_(O), W_5_(IR)* and *W_5_(U)* that the possibilities of *SSP_5_* in the outer-race defect, other bearing faults and the unknown states can also be obtained, respectively.

In Figure 14 *P(I), P(R)* and P*(U)* are the possibility functions of the inner-race defect, the rolling element defect and the unknown states, respectively. Using the matching method explained in Section 5.2, *W_1_(I), W_1_(R)* and *W_1_(U)* that the possibilities of *SSP_1_* in the inner-race defect, the rolling element defect and the unknown states can be obtained, respectively; *W_2_(I), W_2_(R)* and *W_2_(U)* that the possibilities of SSP_2_ in the inner-race defect, the rolling element defect and the unknown states can also be obtained, respectively.

After obtaining the possibilities of the SSPs for each diagnostic step, the combination possibility function of each state *W(N)*′, *W(B)*′, *W(O)*′, *W(IR)*′, *W(I)*′, *W(R)*′ and *W(U)*′ can be obtained by the Dempster & Shafer theory. As an example, parts of training data for each diagnosis step are shown in Tables 6–8.

In order to verify the diagnostic capability of the PLNN, we used the data measured in each state had not been learned by the PLNN. When inputting the test data into the learnt PLNNs, they can correctly and quickly diagnose those faults with the possibility grades of the corresponding states. The diagnosis results are shown in Tables 9–11.

According to the diagnosis results above, the normal (N), the outer-race defect (O), the inner-race defect (I), and the roller element defect (R) states of roller bearing can be automatically and correctly identified using the diagnosis methods proposed in this paper.

## 8. Conclusions

In order to solve the problem of ambiguity between the symptom parameters and fault types, effectively diagnose faults and automatically identify the condition of a rotating machine, an intelligent diagnosis method was proposed on the basis of the least squares mapping (LSM) and a fuzzy neural network. The main conclusions can be summarized as follows:

1. A sequential diagnosis method was proposed through which the fuzzy neural network realized by the partially-linearized neural network (PLNN) could sequentially distinguish fault types.
2. Knowledge for training the PLNN was acquired by possibility theory and the Dempster & Shafer theory (DST). The method of establishing the membership function by converting the probability distribution function of symptom parameters into a possibility function by the possibility theory was proposed, and the combination possibility functions of several symptom parameters were obtained by the DST.
3. The eight non-dimensional symptom parameters in the time domain were defined for reflecting the features of vibration signals measured in each state. To raise the diagnosis sensitivity of the symptom parameters, the new synthetic symptom parameters (SSPs) were obtained by the LSM method.
4. The detection index (DI) on the basis of statistical theory was also defined to evaluate the applicability of the SSPs. The DI can be used to select better SSPs for the PLNN.
5. The practical examples of faults diagnosis of a roller bearing verified the effectiveness of the proposed method. The diagnosis results showed that the faults were sequentially and automatically diagnosed on the basis of the possibilities of the symptom parameters.

## Figures and Tables

**Table 1.** Bearing information for verification.

**Contents**	**Parameters**
Bearing outer diameter	52 mm
Bearing inner diameter	25 mm
Bearing width	15 mm
Bearing roller diameter	7 mm
The number of the rollers	11
Contact angle	0 rad
Outer-race defect	0.3 × 0.25 mm (width × depth); Early stage
Inner-race defect	0.3 × 0.25 mm (width × depth); Early stage
Rolling element defect	0.3 × 0.25 mm (width × depth); Early stage

**Table 2.** Diagnosis sensitivity for condition diagnosis.

**Detection Index**	**Discrimination Rate**	**Sensitivity**
<0.85	<80%	Low
0.85–1.30	80%–90%	Slightly low
1.30–1.65	90%–95%	Middle
1.65–2.33	95%–99%	High
>2.33	>99%	Very high

**Table 3.** Values of DR and DI before projection.

	**P_1_**			**P_2_**		
	
State	μ_p1_	σ_p1_	DI_P1_ (DR_P1_)	μ_p2_	σ_p2_	DI_P2_ (DR_P2_)
I	2.38	0.35	1.12 (86.9%)	0.72	0.17	1.19 (87.3%)
R	3.12	0.56		0.435	0.168	

**Table 4.** Values of DR and DI after projection.

	**SSP_1_**			**SSP_2_**		
	
State	μ_ssp1_	σ_ssp1_	DI_ssp1_ (DR_ssp1_)	μ_ssp2_	σ_ssp2_	DI_ssp2_ (DR_ssp2_)
I	3.99	0.37	2.34 (99.04%)	1.025	0.0022	2.25 (98.8%)
R	5.13	0.32		1.032	0.0022	

**Table 5.** DI values of SSPs for each sequential diagnosis step.

**DI Values of Each SSP**								
	**SSP_1_**	**SSP_2_**	**SSP_3_**	**SSP_4_**	**SSP_5_**	**SSP_6_**	**SSP_7_**	**SSP_8_**
For first step								
N:O	**13.86**	3.11	1.43	2.10	**10.38**	4.93	9.48	7.11
N:I	**2.92**	2.20	1.11	2.39	**3.08**	2.76	2.72	2.56
N:R	**4.81**	3.37	0.77	1.06	**3.43**	1.23	2.27	1.06
For second step								
O:I	**4.69**	0.70	0.88	2.05	**3.62**	2.52	3.31	2.31
O:R	**3.01**	2.41	1.56	0.80	**2.35**	1.04	1.00	0.80
For third step								
I:R	**2.34**	**2.12**	1.22	1.63	1.03	0.70	1.45	1.11

**Table 6.** Training data for first step of sequential diagnosis.

**SSP_1_**	**SSP_5_**	***W*(*N*)′**	***W*(*B*)′**	***W*(*U*)′**
1.245	0	0	0	1
2.76	38.7	0.5	0.02	0.48
5.35	0.665	0.333	0.38	0.287
4.52	6.18	0	1	0
…	…	…	…	…

**Table 7.** Training data for second step of sequential diagnosis.

**SSP_1_**	**SSP_5_**	***W*(*O*)′**	***W*(*IR*)′**	***W*(*U*)′**
3.15	6.17	0	0	1
4.13	6.42	0.333	0.333	0.333
6.08	6.5	0.978	0	0.022
5.04	15.1	0	1	0
…	…	…	…	…

**Table 8.** Training data for third step of sequential diagnosis.

**SSP_1_**	**SSP_2_**	***W*(*I*)′**	***W*(*R*)′**	***W*(*U*)′**
2.5	1.01	0	0	1
3.835	1.021	0.75	0	0.25
5.332	1.021	0.333	0.333	0.333
5.66	1.032	0.057	0.943	0
…	…	…	…	…

**Table 9.** Verification result of first step.

**SSP_1_**	**SSP_5_**	***W*(*N*)′**	***W*(*B*)′**	***W*(*U*)′**	**Judge**
3.025	1.854	0.811	0.112	0.105	N
2.882	1.615	0.796	0.157	0.138	N
4.260	26.05	0.0002	0.8405	0.1691	B
4.961	15.53	0.0002	0.8561	0.1462	B
1.579	30.56	0.036	0.0928	0.9075	U
…	…	…	…	…	…

**Table 10.** Verification result of second step.

**SSP_1_**	**SSP_5_**	***W*(*O*)′**	***W*(*IR*)′**	***W*(*U*)′**	**Judge**
6.10	6.33	0.8607	0.0021	0.1511	O
6.104	6.84	0.9105	0.0059	0.1023	O
4.22	18.44	0.1265	0.8365	0.0732	I or R
5.36	9.93	0.0671	0.8012	0.1747	I or R
2.01	25.5	0.1011	0.0936	0.8228	U
…	…	…	…	…	…

**Table 11.** Verification result of third step.

**SSP_1_**	**SSP_2_**	***W*(*I*)′**	***W*(*R*)′**	***W*(*U*)′**	**Judge**
3.81	1.025	0.9541	0.0035	0.1231	I
4.09	1.029	0.9027	0.0071	0.1096	I
5.26	1.031	0.0082	0.8974	0.1217	R
4.73	1.033	0.0047	0.9127	0.1056	R
6.69	0.83	0.0767	0.0458	0.9279	U
…	…	…	…	…	…
